# Development and validation of nomograms for predicting depression and suicidal ideation in stroke survivors: a community-based study

**DOI:** 10.1186/s12888-025-07767-3

**Published:** 2026-01-07

**Authors:** Zicheng Cheng, Lili Ma, Meiqi Zhao, Tong Xu, Jianing Wang, Xinxia Zhu, Zhao Han

**Affiliations:** Department of Neurology, The Second Affiliated Hospital and Yuying Children’s Hospital of Wenzhou Medical University, Wenzhou, Zhejiang Province 325000 China

**Keywords:** Community, Depression, Nomogram, Prediction, Primary healthcare, Stroke, Suicidal ideation

## Abstract

**Background:**

Stroke is a major global health burden, with post-stroke depression and suicidal ideation prevalent yet often underdiagnosed complications. Existing prediction models rely on acute-phase, hospital-based data, limiting their applicability in community settings. This study aimed to develop community-applicable nomograms for predicting risk of depression and suicidal ideation in stroke survivors.

**Methods:**

Using data from the National Health and Nutrition Examination Survey (NHANES, 2005–2018), predictors included sociodemographic, lifestyle, medical history, functional status, and blood test variables. Lasso regression and the Boruta algorithm were used for predictor selection. Logistic regression models were developed, and nomograms were constructed. Prediction model performance was evaluated through discrimination (area under curve [AUC]), calibration (calibration plot and Brier score), and clinical utility (decision curve analysis). Internal validation used bootstrapping, and external validation was performed on temporally distinct NHANES datasets.

**Results:**

The prevalence of depression among stroke survivors was 34.8%, with one-third of cases going unrecognized or untreated. The prevalence of suicidal ideation among stroke survivors was 8.2%. The nomograms demonstrated strong discriminative ability (AUC: 0.76 for depression, 0.74 for suicidal ideation) and calibration accuracy (Brier score: 0.11 for depression, 0.07 for suicidal ideation). Key predictors for depression included age, marital status, current smoking, cancer, healthcare utilization, sleep duration, and inability to work. For suicidal ideation, marital status, hypertension, arthritis, antidepressant use, and inability to work were significant. Decision curve analysis demonstrated clinical utility within probability thresholds of 6%–40% for depression and 3%–17% for suicidal ideation. Internal and external validation supported generalizability and reproducibility of the nomograms.

**Conclusions:**

This study provides validated, community-applicable nomograms for predicting depression and suicidal ideation in stroke survivors, enabling primary care providers to identify high-risk individuals and facilitate timely mental health interventions.

**Clinical trial number:**

Not applicable.

**Supplementary Information:**

The online version contains supplementary material available at 10.1186/s12888-025-07767-3.

## Introduction

Stroke represents a mounting global health challenge, with an annual incidence rate of 141.6 per 100,000 individuals worldwide in 2021 [1]. Alarming projections estimate that stroke survivors will increase to 159.3 million by 2050, driven by aging populations, persistent risk factors, and socioeconomic disparities across regions [2]. Concurrently, stroke imposed a $2.06 trillion global economic burden in 2019, highlighting the urgent need for optimized long-term health management for stroke survivors [3]. Mood disorders are common and serious complications of stroke. Systematic reviews indicate that over half of stroke survivors develop depression during the disease course, with cumulative incidence reaching 55% within 15 years, while 12.2% of survivors report suicidal ideation [4]. These psychological consequences demand urgent clinical and public health attention, as early identification of post-stroke depression not only enhances functional recovery and quality of life but also significantly improves survival outcomes [5].

Although significant advancements have been made in developing prediction models for post-stroke depression, significant limitations remain in their real-world applicability. Existing models predominantly focus on acute care populations, predicting depression risk from hospital discharge to 6-month post-stroke using hospitalization-based indicators (e.g., National Institute of Health stroke scale [NIHSS] scores, acute-phase biomarkers including blood tests and brain magnetic resonance imaging [MRI] features) [6–13]. While these hospital-centric algorithms demonstrate strong predictive validity in controlled settings (area under curve [AUC]: 0.70 − 0.93), their reliance on specialized neurological indices creates a fundamental implementation barrier for chronic stroke management in community ​settings. This discrepancy highlights a critical gap, as no validated tools currently exist for predicting depression risk in community-dwelling stroke survivors. The absence of community-applicable models utilizing routinely available primary healthcare data, ​such as socioeconomic features, functional status, and lifestyle patterns, ​substantially impedes preventive mental health interventions in non-specialist settings. Building upon existing prediction models for post-stroke depression in hospitalized patients during the acute phase, developing a depression prediction model for community-dwelling stroke survivors can enable more comprehensive and continuous management of mental health throughout the stroke care continuum.

Compared to the numerous models for post-stroke depression, prediction models specifically designed for post-stroke suicidal ideation are remarkably scarce. This neglect carries grave implications, ​given the 1.4 − 4.5 fold increased risk of suicidal ideation among stroke survivors compared to the general population [14, 15]. Approximately 3 to 4 per 1000 stroke survivors die by suicide within the first five years after stroke [4]. According to ideation-to-action theories of suicide, suicidal ideation precedes suicide attempts and may ultimately lead to completed suicide [16]. Early identification through predictors of suicidal ideation accessible in community ​settings could potentially mitigate suicide mortality risks in this vulnerable population.

Nomograms combine multiple predictors to generate an individual probability of a clinical event, improving accuracy and usability. Their intuitive and user-friendly digital interfaces support seamless integration into clinical decision making, advancing personalized medicine [17]. To bridge these gaps, therefore, we aimed to develop the community-applicable nomograms predicting depression and suicidal ideation risks in stroke survivors. Distinct from prior models requiring specialized neurological assessments, our nomograms were designed based on data that is routinely obtained through brief interviews and preliminary physical examinations in primary healthcare. The purpose of the nomograms is to assist primary care physicians or providers in screening community-dwelling stroke survivors for individuals at high risk of depression and suicidal ideation, thereby facilitating their referral to psychiatric specialists for further evaluation and intervention.

## Methods

### Study population

**Fig. 1:**
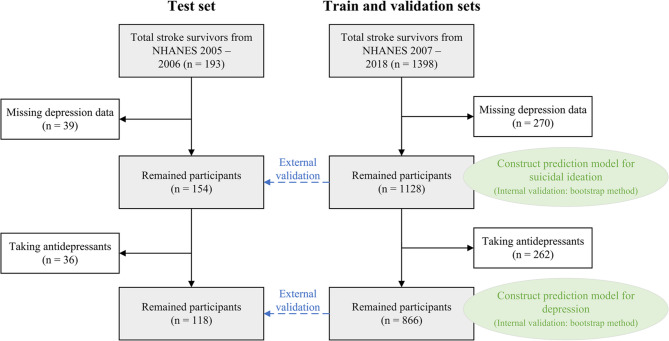
Flowchart of participant selection

Individual participant data from the National Health and Nutrition Examination Survey (NHANES) between 2005 and 2018 were used. The NHANES was designed to evaluate the health and nutritional status of the general population in the United States [18]. The survey procedures were approved by the National Center for Health Statistics Ethics Review Board, and all participants provided informed consents. Participants completed a medical conditions interview asked by trained interviewers. Stroke survivors were identified by a “yes” answer to “Has a doctor or other health professional ever told you that you had a stroke?“. We excluded participants who did not receive a complete depression scale assessment. The study flowchart is presented in Fig. 1. In the development and validation of prediction model for depression among stroke survivors, participants taking antidepressant medications were excluded. On the one hand, this exclusion criterion was implemented to ensure the model’s suitability for the purpose of preliminary depression screening. On the other hand, the antidepressant use might alter the manifestation of correlates of depression. This study followed the Transparent Reporting of a Multivariable Prediction Model for Individual Prognosis or Diagnosis plus artificial intelligence (TRIPOD + AI) reporting guideline [19].

### Data collection

The selection of study variables was based on influential factors documented in previous literature, while simultaneously considering the availability of corresponding indicators in the NHANES database and ensuring feasibility of data collection in primary healthcare settings. The integrated framework of the biopsychosocial model emphasizes the three-dimensional risk factors of biological, psychological, and social aspects in disease causation [20]. Thus, the socioeconomic features, lifestyle patterns, disease and medical conditions, functional status, as well as simple blood test data of participants were collected from the database. Sociodemographic features included age, sex (female, male), race/ethnicity (Mexican American, other Hispanic, non-Hispanic White, non-Hispanic Black, other race), marital status (married/living with partner, widowed/divorced/separated, never married), education level (less than high school, high school, more than high school), and the family income-to-poverty ratio (PIR) (< 1.3, 1.3 − 3.5, > 3.5). Lifestyle patterns contained current smoking, drinking, physical inactivity, sedentary time, and sleep duration (< 7 h, 7–9 h, > 9 h). Disease and medical conditions were composed of body mass index, waist-to-height ratio, hypertension, diabetes mellitus, hyperlipidemia, cardiovascular diseases, arthritis, cancer, hemodialysis, medical insurance, hospitalization and number of healthcare in the past year, and antidepressant use. Functional status focused on whether the participants’ work capacity was limited. Simple blood test included albumin and neutrophil-to-lymphocyte ratio. The detailed definitions of the indicators for lifestyle patterns and disease and medical conditions are listed in Supplementary Table 1.

### Outcome

The nine-item Patient Health Questionnaire (PHQ-9) was administered to assess depression and suicidal ideation in stroke survivors. Depression was diagnosed when the PHQ-9 score ≥ 10 [21]. For the PHQ-9 item nine: " Over the last two weeks, how often have you felt that you would be better off dead, or hurting yourself in some way?“, participants who responded affirmatively were identified as having suicidal ideation [22, 23].

### Statistical analysis

#### Model development

**Fig. 2:**
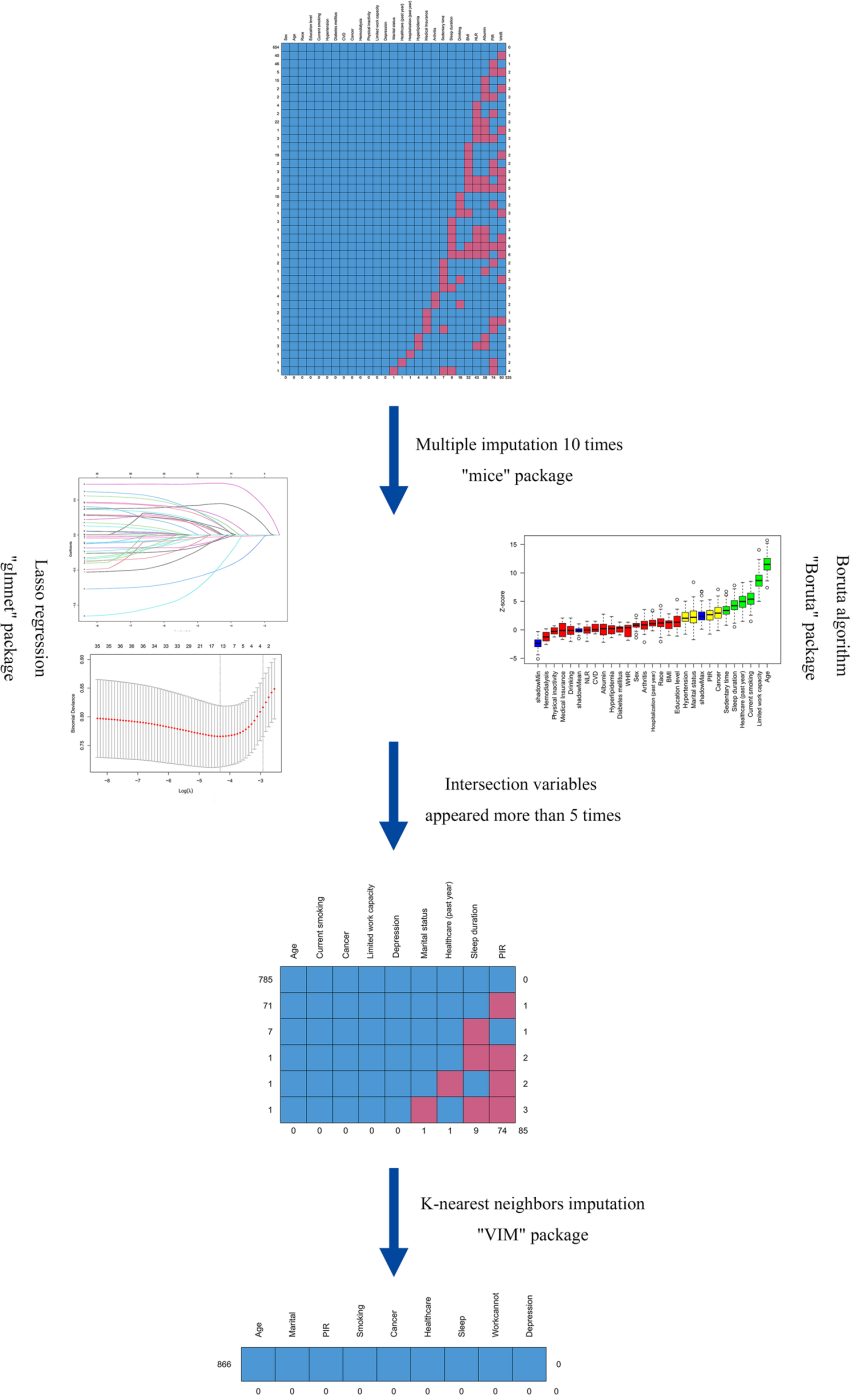
Flowchart of variable imputation and selection

Statistical analysis was performed using R statistical software (version 4.4.2). In the preliminary selection of outcome predictors, this study first addressed missing data using multiple imputation. The ‘mice’ package was employed to generate 10 complete datasets, with variables having a missing rate exceeding 20% pre-set to be excluded from the imputation process. In practice, the variable with the most missing values was the waist-to-height ratio, accounting for ​​80/866 (9.2%)​​ in the depression dataset and ​​120/1128 (10.6%)​​ in the suicidal ideation dataset. Since neither exceeded the 20% cutoff, ​​all missing variables underwent multiple imputation​​. Subsequently, least absolute shrinkage and selection operator (Lasso) regression and the Boruta algorithm were applied to each imputed dataset for variable screening, and the intersection of the results from both methods was taken. In Lasso regression, variables were selected based on the model corresponding to the lambda value that minimized the mean squared error. For the Boruta algorithm, variables were retained if their importance Z-Score exceeded that of shadow variables or if their importance score was comparable to shadow variables. Finally, variables that appeared in 8 or more out of the 10 datasets were selected for inclusion in the subsequent logistic regression model. Prior to modeling, the missing value patterns of the selected variables in the original data were examined, and the K-nearest neighbors (KNN) algorithm was used to impute missing values, generating a complete dataset suitable for modeling. Figure 2 illustrates the aforementioned process. Finally, stepwise logistic regression was performed, and the model with the smallest Akaike Information Criterion (AIC) was selected to construct the visualized nomogram. The variance inflation factor (VIF) was employed to evaluate multicollinearity between independent variables included in the model, where VIF > 10 suggested significant multicollinearity.

#### Model validation

The statistical performance of the prediction model was evaluated from three aspects: discrimination, calibration, and clinical utility. Discrimination was assessed using the area under curve (AUC) of the receiver operating characteristic (ROC) curve, reflecting the model’s ability to distinguish between stroke survivors with and without depression or suicidal ideation. Calibration was evaluated using calibration plot and the Brier score, reflecting the consistency between the model’s predicted risks and the observed risks in the data. Clinical utility was assessed using decision curve analysis (DCA) to measure the model’s practical value in clinical decision-making by quantifying the net benefits across a range of threshold probabilities [24]. The NHANES 2007 − 2018 dataset was used as the training set to develop the prediction model and construct the nomogram. Internal validation was performed using the bootstrap method, which involved 1000 resampling iterations to generate 1000 training sets, with the original training set serving as the validation set to assess the model’s optimism and statistical performance. External validation was conducted on the NHANES 2005 − 2006 dataset as a temporal validation to evaluate the model’s generalizability across different time periods.

## Results

### Baseline characteristics of study participants

The training and validation datasets were derived from NHANES 2007 − 2018, including 1128 stroke survivors with complete PHQ-9 assessments, of whom 262 (23.2%) were taking antidepressants. The depression prediction model was developed using data from 866 participants (15.0% with depression; 45.6% female; mean age: 68.0 years, interquartile range [IQR]: 58.0–77.0), whereas the suicidal ideation prediction model utilized data from all 1128 participants (8.2% with suicidal ideation; 50.9% female; mean age: 67.0 years, IQR: 57.5–77.0). Clinical characteristics of these participants are summarized in Table 1. For external validation, the test set was derived from NHANES 2005 − 2006, including 154 stroke survivors with complete PHQ-9 assessments, of whom 36 (23.4%) were taking antidepressants. The depression prediction model was validated using data from 118 participants (13.6% with depression; 45.8% female; mean age: 69.0 years, interquartile range [IQR]: 61.0–80.0), while the suicidal ideation prediction model was validated using data from all 154 participants (9.1% with suicidal ideation; 48.7% female; mean age: 68.0 years, IQR: 61.0–78.0). Clinical characteristics of the test set participants are provided in Supplementary Table 2.

**Table 1:** Participant characteristics in the NHANES 2007 and 2018

Characteristic	Data for depression			Data for suicidal ideation		
	Overall (*n* = 866)	Without depression (*n* = 736)	With depression (*n* = 130)	Overall (*n* = 1128)	Without suicidal ideation (*n* = 1036)	With suicidal ideation (*n* = 92)
**Socioeconomic features**						
Age, years	68.0 (58.0–77.0)	68.0 (60.0–78.0)	60.0 (49.0–71.0)	67.0 (57.5–77.0)	68.0 (58.0–77.0)	63.0 (56.0–73.0)
Sex, %						
Female	395 (45.6)	326 (44.3)	69 (53.1)	574 (50.9)	517 (49.9)	57 (62.0)
Male	471 (54.4)	410 (55.7)	61 (46.9)	554 (49.1)	519 (50.1)	35 (38.0)
Race, %						
Mexican American	80 (9.2)	66 (9.0)	14 (10.8)	95 (8.4)	84 (8.1)	11 (12.0)
Other Hispanic	58 (6.7)	49 (6.7)	9 (6.9)	72 (6.4)	65 (6.3)	7 (7.6)
Non-Hispanic White	390 (45.0)	337 (45.8)	53 (40.8)	561 (49.7)	508 (49.0)	53 (57.6)
Non-Hispanic Black	268 (30.9)	226 (30.7)	42 (32.3)	314 (27.8)	298 (28.8)	16 (17.4)
Other Race	70 (8.1)	58 (7.9)	12 (9.2)	86 (7.6)	81 (7.8)	5 (5.4)
Education level, %						
Less than high school	295 (34.1)	236 (32.1)	59 (45.4)	368 (32.6)	330 (31.9)	38 (41.3)
High school	244 (28.2)	216 (29.3)	28 (21.5)	321 (28.5)	295 (28.5)	26 (28.3)
More than high school	327 (37.8)	284 (38.6)	43 (33.1)	439 (38.9)	411 (39.7)	28 (30.4)
Marital status, %						
Married/living with partner	457 (52.8)	406 (55.2)	51 (39.5)	577 (51.2)	544 (52.6)	33 (35.9)
Widowed/divorced/separated	325 (37.6)	268 (36.4)	57 (44.2)	446 (39.6)	403 (38.9)	43 (46.7)
Never married	83 (9.6)	62 (8.4)	21 (16.3)	104 (9.2)	88 (8.5)	16 (17.4)
PIR, %						
< 1.3	328 (41.4)	263 (39.1)	65 (54.6)	428 (41.3)	381 (40.1)	47 (54.7)
1.3–3.5	327 (41.3)	284 (42.2)	43 (36.1)	429 (41.4)	400 (42.1)	29 (33.7)
> 3.5	137 (17.3)	126 (18.7)	11 (9.2)	179 (17.3)	169 (17.8)	10 (11.6)
**Lifestyle patterns**						
Current smoking, %	211 (24.4)	155 (21.1)	56 (43.1)	289 (25.6)	256 (24.7)	33 (35.9)
Drinking, %	511 (60.1)	423 (58.7)	88 (68.2)	661 (59.7)	601 (59.1)	60 (65.9)
Physical inactivity, %	518 (59.8)	435 (59.1)	83 (63.8)	696 (61.7)	623 (60.1)	73 (79.3)
Sedentary time, minutes	360.0 (240.0–480.0)	360.0 (240.0–480.0)	360.0 (240.0–540.0)	360.0 (240.0–480.0)	360.0 (240.0–480.0)	360.0 (180.0–540.0)
Sleep duration, %						
< 7 h	308 (35.9)	243 (33.2)	65 (52.0)	391 (35.0)	353 (34.3)	38 (42.7)
7–9 h	479 (55.9)	432 (59.0)	47 (37.6)	606 (54.3)	570 (55.4)	36 (40.4)
> 9 h	70 (8.2)	57 (7.8)	13 (10.4)	120 (10.7)	105 (10.2)	15 (16.9)
**Disease and medical conditions**						
Body mass index, %						
< 18.5 kg/m^2^	13 (1.6)	10 (1.4)	3 (2.4)	16 (1.5)	14 (1.4)	2 (2.4)
18.5–24.9 kg/m^2^	208 (24.9)	171 (24.2)	37 (29.4)	245 (22.7)	228 (22.9)	17 (20.0)
25.0–29.9 kg/m^2^	254 (30.5)	224 (31.6)	30 (23.8)	331 (30.6)	305 (30.7)	26 (30.6)
≥ 30.0 kg/m^2^	359 (43.0)	303 (42.8)	56 (44.4)	488 (45.2)	448 (45.0)	40 (47.1)
Waist-to-height ratio, %						
< 0.5	58 (7.4)	49 (7.3)	9 (7.6)	70 (6.9)	64 (6.9)	6 (7.7)
≥ 0.5	728 (92.6)	619 (92.7)	109 (92.4)	938 (93.1)	866 (93.1)	72 (92.3)
Hypertension, %	688 (79.4)	584 (79.3)	104 (80.0)	903 (80.1)	834 (80.5)	69 (75.0)
Diabetes mellitus, %	332 (38.3)	284 (38.6)	48 (36.9)	444 (39.4)	401 (38.7)	43 (46.7)
Hyperlipidemia, %	587 (68.1)	496 (67.7)	91 (70.5)	769 (68.5)	704 (68.2)	65 (72.2)
Cardiovascular diseases, %	315 (36.4)	254 (34.5)	61 (46.9)	420 (37.2)	376 (36.3)	44 (47.8)
Arthritis, %	445 (51.7)	368 (50.3)	77 (59.7)	644 (57.3)	575 (55.7)	69 (75.8)
Cancer, %	182 (21.0)	150 (20.4)	32 (24.6)	249 (22.1)	225 (21.7)	24 (26.1)
Hemodialysis, %	11 (1.3)	9 (1.2)	2 (1.5)	15 (1.3)	13 (1.3)	2 (2.2)
Medical insurance, %	767 (89.0)	655 (89.4)	112 (86.8)	1015 (90.4)	932 (90.4)	83 (90.2)
Hospitalization in the past year, %	284 (32.8)	235 (32.0)	49 (37.7)	371 (32.9)	337 (32.6)	34 (37.0)
Number of healthcare in the past year, %						
0	38 (4.4)	31 (4.2)	7 (5.4)	41 (3.6)	37 (3.6)	4 (4.3)
1	57 (6.6)	54 (7.3)	3 (2.3)	69 (6.1)	67 (6.5)	2 (2.2)
≥ 2	770 (89.0)	651 (88.5)	119 (92.2)	1017 (90.2)	931 (90.0)	86 (93.5)
Antidepressant use, %	—	—	—	262 (23.2)	226 (21.8)	36 (39.1)
**Functional status**						
Limited work capacity, %	376 (43.4)	286 (38.9)	90 (69.2)	535 (47.4)	464 (44.8)	71 (77.2)
**Blood tests**						
Albumin, mg/L	4.1 (3.9–4.3)	4.1 (3.9–4.4)	4.1 (3.9–4.3)	4.1 (3.9–4.3)	4.1 (3.9–4.3)	4.1 (3.9–4.3)
Neutrophil-to-lymphocyte ratio	2.1 (1.6–2.9)	2.1 (1.6–2.9)	2.1 (1.5–3.1)	2.2 (1.6–3.1)	2.2 (1.6–3.1)	2.3 (1.6–3.3)

PIR, family income-to-poverty ratio

### Model specification

For each dataset generated through multiple imputation, variable selection was performed using both Lasso regression and the Boruta algorithm, with the final variables determined by their intersection. The variables consistently selected for the depression prediction model, along with their selection frequencies across imputed datasets, were as follows: age (10), marital status (9), PIR (7), current smoking (10), cancer (10), number of healthcare in the past year (10), sleep duration (10), and inability to work (10). For the suicidal ideation prediction model, the selected variables and their frequencies were: age (10), education level (1), marital status (10), current smoking (2), hypertension (8), arthritis (10), number of healthcare in the past year (8), sedentary time (8), inability to work (8), and antidepressant use (9). Variables with a selection frequency ≥ 8 were included in the logistic regression model, and further refined using stepwise regression. The results of the multivariate logistic regression are presented in Table 2. For the depression prediction model, the final selected variables included age, marital status, current smoking, sleep duration, cancer, number of healthcare in the past year, and inability to work. For the suicidal ideation prediction model, the significant predictors were marital status, hypertension, arthritis, antidepressant use, and inability to work.

**Table 2:** Multivariable predictors of depression and suicidal ideation among stroke survivors in the NHANES 2007 and 2018

Characteristic	Depression		Suicidal ideation	
	OR (95% CI)	*p*-value	OR (95% CI)	*p*-value
Age, years	0.97 (0.95–0.98)	< 0.001	—	—
Marital status				
Married/living with partner	Ref		Ref	
Widowed/divorced/separated	1.75 (1.13–2.71)	0.012	1.53 (0.94–2.49)	0.086
Never married	1.65 (0.86–3.09)	0.12	2.53 (1.28–4.86)	0.006
Current smoking	1.84 (1.18–2.85)	0.007	—	—
Cancer	1.61 (0.98–2.60)	0.056	—	—
Number of healthcare in the past year			—	—
0	2.94 (0.70–15.2)	0.16		
1	Ref			
> 1	3.64 (1.23–15.8)	0.040		
Sleep duration			—	—
< 7 h	2.15 (1.41–3.30)	< 0.001		
7–9 h	Ref			
> 9 h	1.67 (0.78–3.38)	0.17		
Limited work capacity	2.55 (1.68–3.93)	< 0.001	3.45 (2.09–5.91)	< 0.001
Hypertension	—	—	0.58 (0.35–1.00)	0.045
Arthritis	—	—	1.94 (1.17–3.32)	0.012
Antidepressant use	—	—	1.69 (1.05–2.69)	0.028

CI, confidence interval; OR, odds ratio

### Model development and validation

**Fig. 3:**
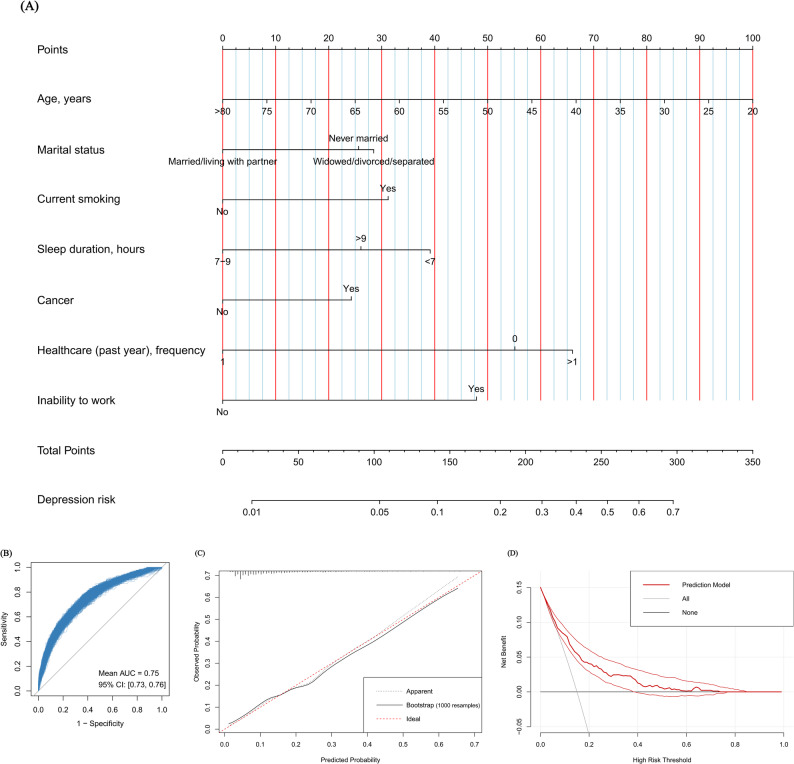
Nomogram for predicting depression risk in stroke survivors and its internal validation results using bootstrap resampling with 1000 iterations. (**A**): nomogram; (**B**): receiver operating characteristic curve; (**C**): calibration plot; (**D**): decision curve analysis

Figure 3A presents the nomogram developed to estimate depression risk in stroke survivors. The nomogram integrates multiple predictors weighted by their regression coefficients, where the summation of assigned points maps to a probability scale for clinical interpretation. An AUC of 0.76 indicated strong discriminative ability, and an AUC of 0.75 (95% confidence interval [CI]: 0.73–0.76) in bootstrapped validation indicated minimal optimism (Fig. 3B; Supplementary Fig. 1A). Calibration plot showed strong agreement between predicted and observed outcomes (Fig. 3C), supported by a Brier score of 0.11 (95% CI: 0.11–0.11) (Supplementary Fig. 1B). DCA further validated clinical utility, showing superior net benefit over “treat-all” and “treat-none” approaches across a clinically relevant probability threshold range (6%–40%) (Fig. 3D). External validation reinforced model generalizability, achieving an AUC of 0.78, a Brier score of 0.10, and well-performed calibration plot and DCA result (Supplementary Fig. 2).

**Fig. 4:**
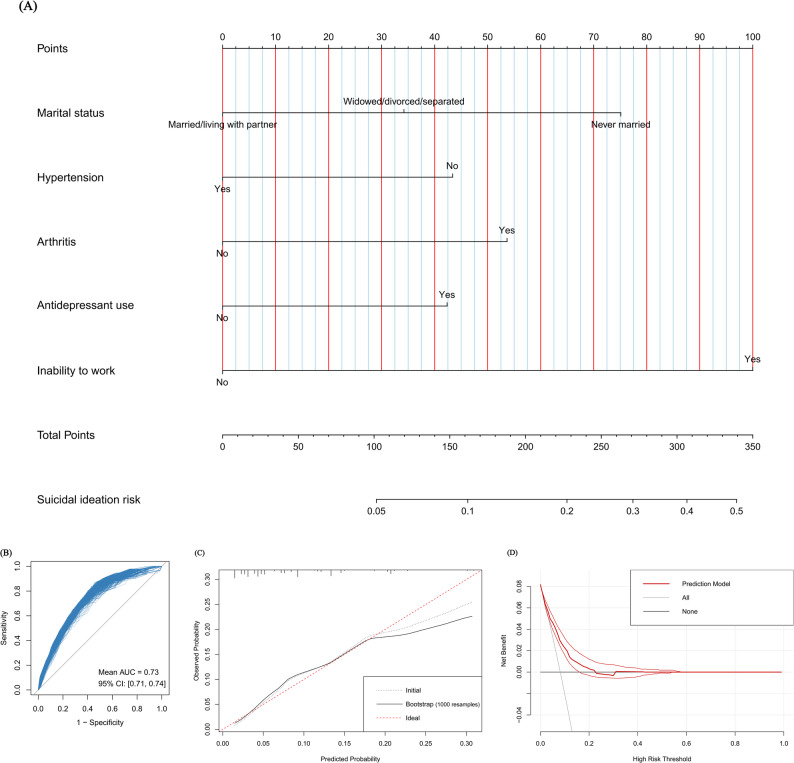
Nomogram for predicting suicidal ideation risk in stroke survivors and its internal validation results using bootstrap resampling with 1000 iterations. (**A**): nomogram; (**B**): receiver operating characteristic curve; (**C**): calibration plot; (**D**): decision curve analysis

The nomogram for estimating suicidal ideation risk in stroke survivors is illustrated in Fig. 4A. The discrimination was evidenced by an AUC of 0.74, with internal validation maintaining an AUC of 0.73 (95% CI: 0.71–0.74), suggesting negligible optimism (Fig. 4B; Supplementary Fig. 3A). Calibration accuracy was confidence determined through calibration plot (Fig. 4C) and a low Brier score (0.07; 95% CI: 0.07–0.07) (Supplementary Fig. 3B). DCA demonstrated meaningful clinical applicability within the 3%–17% probability threshold range (Fig. 4D). External validation metrics, including an AUC of 0.80, a Brier score of 0.08, calibration plot, and DCA result, confirmed satisfactory model reproducibility (Supplementary Fig. 4).

## Discussion

This study developed and validated visualized nomograms for predicting depression and suicidal ideation risk among stroke survivors based on data from NHANES 2007 − 2018 and 2005 − 2006. The results showed that the nomograms had strong discriminative ability and calibration accuracy. Key predictors for the depression model included age, marital status, current smoking, cancer, number of healthcare in the past year, sleep duration, and inability to work, while the suicidal ideation model highlighted marital status, hypertension, arthritis, inability to work, and antidepressant use as significant predictors. Furthermore, DCA confirmed the clinical utility of the nomograms when probability thresholds set at 6%–40% for depression and 3%–17% for suicidal ideation.

In the NHANES 2007–2018, approximately one-quarter of stroke survivors received antidepressant treatment. Among those not taking antidepressants, 15% still experienced depression. Moreover, 8.2% of stroke survivors reported suicidal ideation. These findings align with previous studies [4], highlighting a significantly higher incidence of post-stroke depression and suicidal ideation. This phenomenon is associated with multiple pathophysiological mechanisms, including hypothalamic-pituitary-adrenal axis dysfunction, enhanced inflammatory responses, neurotransmitter abnormalities, and reduced neurotrophic factor levels [25]. The high prevalence of undiagnosed depression and suicidal ideation among stroke survivors underscores the urgent need to develop user-friendly prediction models for identifying high-risk individuals in community settings. Although several prediction models for post-stroke depression have been proposed, with the top-performing model achieving an AUC of 0.93, significant limitations remain [11]. First, the model incorporates predictors that are difficult to obtain in community settings, such as stroke location, NIHSS score, and methylation levels at the cg02550950 site. Second, predictors and depression diagnoses were collected at different time points, limiting its utility for cross-sectional community screening. Additionally, for post-stroke suicidal ideation, only one machine learning model has been developed based on 385 stroke patients. However, this model lacks interpretability and external validation, potentially hindering its practical application [26]. To address these limitations, we rigorously developed and validated prediction models in this community-based study. To optimize predictor selection and model performance, we employed a comprehensive approach combining Lasso regression, the Boruta algorithm, and stepwise regression. This multi-method strategy effectively balanced simplicity and predictive accuracy while minimizing overfitting. Both internal and external validation procedures demonstrated robust model performance, with low optimism, excellent discrimination, and satisfactory calibration, highlighting its potential clinical utility. This study provides a valuable tool and reference for mental health management in stroke survivors.

The predictors in our prediction model of post-stroke depression align with previous studies, emphasizing the critical role of sociodemographic factors, health behaviors, and chronic conditions in mental health risk. Younger stroke survivors are more susceptible to post-stroke depression, which is linked to subjective stress, possibly because they face greater pressure in terms of social roles, financial burdens, and family responsibilities [27]. Individuals who are widowed, divorced, or separated have an increased risk of depression after stroke, attributable to the absence of emotional support and life assistance provided by a spouse [28]. Both smoking and short sleep duration are closely associated with post-stroke depression, not only because they can induce depressive symptoms, but also because depression exacerbates smoking behavior and insomnia due to increased psychological stress [29, 30]. The prevalence of depression among cancer patients is significantly higher than that in the general population, ranging from 1.5% to 50%, with an average prevalence rate of 15% to 29% [31]. This elevated risk is attributed to the interplay of multiple psychological and physiological factors associated with cancer and its treatment. As demonstrated in previous studies, post-stroke depression is associated with increased healthcare utilization, characterized by elevated number of hospital admissions, outpatient visits, and cumulative length of hospital stay [32]. Inability to work, which reflects severe neurological deficits following stroke, contributes to the concerns about economic instability and social isolation in stroke patients, subsequently leading to depression [33].

Compared to prediction models for post-stroke depression, models for post-stroke suicidal ideation are relatively scarce. Most of the predictors incorporated in our model are generally consistent with previous research, while further emphasizing the significant role of psychosocial and physical health in post-stroke suicidal ideation. Marital status and inability to work, as common predictors in both depression prediction models and suicidal ideation prediction models, indicate that psychological factors constitute a significant pathogenic mechanism in mental disorders [34]. Arthritis increases the risk of suicidal ideation by 1.5 times due to restricted physical activity and chronic pain [35]. Note that predictive relationships do not imply causation. Prediction research aims to identify a minimal set of variables associated with an outcome to maximize predictive accuracy, regardless of whether these variables are causally related. This consideration should be noted when interpreting hypertension and antidepressant use as predictors of post-stroke suicidal ideation. The association of hypertension and antidepressant use with post-stroke suicidal ideation may be indirect or confounded. For hypertension, potential explanations may involve the effects of antihypertensive medications [36, 37], as well as the possibility that hypertensive patients pay more attention to their health and actively seek healthcare services. The use of antidepressant medication indicates the presence of depression, and the risk of suicidal ideation in individuals with post-stroke depression is 2.3 times higher than in those without depression [38].

DCA offers guidance on the application of nomograms on the basis of the threshold probability, that triggers a medical intervention by a physician or patient. Clinical benefits can be achieved by intervening in individuals with a depression risk greater than 6%–40% or a suicidal ideation risk greater than 3%–17%. Since the nomograms are designed to screen high-risk individuals for depression or suicidal ideation among stroke survivors rather than for diagnostic purposes, and facilitating further psychiatric consultation for high-risk individuals is a non-harmful intervention, we consider it is reasonable to set the threshold probability near the lower end of the range. Primary care providers can use verbal interviews to gather key indicators and apply the nomogram to calculate the probability of depression or suicidal ideation, thereby enabling timely referral of high-risk individuals to psychiatrists for definitive diagnosis and treatment, particularly in resource-limited settings with inadequate mental health infrastructure.

To the best of our knowledge, this is the first community-based study to develop and validate prediction models for depression and suicidal ideation in stroke survivors suitable for primary healthcare. However, several limitations of our study need to be addressed. First, depression and suicidal ideation were determined based on participants’ responses to the PHQ-9. Stroke survivors with dementia or speech disorders were unable to complete the scale assessment, which limits the application of our model to these patients. Secondly, although external validation indicates the stable statistical performance of the models, this was conducted through temporal validation in datasets with similar participant characteristics. Geographic validation based on stroke survivors from different regions is still required to demonstrate the model’s transportability. Third, the lack of subgroup performance metrics (e.g., by sex, race, or stroke type) limits insight into the model’s generalizability. Subsequent external validation should evaluate model performance across clinically relevant subgroups through prespecified stratified analyses. Fourth, the advancement of artificial intelligence has expanded the diagnostic tools for mental disorders, including the use of natural language processing and large language models to analyze speech samples, electronic medical records, and social media data [39, 40]. Integrating artificial intelligence technologies with traditional predictors in future models could enhance the precision of depression and suicide risk screening in primary healthcare.

## Conclusions

In conclusion, we have identified easily accessible predictors for constructing prediction models for depression and suicidal ideation in stroke survivors. The predictive performance of the prediction models is good. Visualized nomograms enables primary care physicians or non-neurological and non-psychiatric specialists to estimate the risk of post-stroke depression and suicidal ideation in community settings. This bridges the critical gap between community care and specialist mental health services for stroke survivors, ensuring timely intervention through risk-stratified pathways, particularly valuable in regions with limited specialist access.

## Supplementary Information

Below is the link to the electronic supplementary material.

Supplementary Material 1

## Data Availability

The datasets generated and analysed during the current study are available from the corresponding author on reasonable request.
