# Supplementary material for: Development and validation of nomograms for predicting depression and suicidal ideation in stroke survivors: a community-based study
Source: BMC Psychiatry. 2026 Jan 7;26:117. doi: 10.1186/s12888-025-07767-3 (PMC12870091; doi:10.1186/s12888-025-07767-3)
Supplement: Supplementary file 1 — Supplementary Material 1 [file 12888_2025_7767_MOESM1_ESM.docx]

**Development and validation of nomograms for predicting depression and suicidal ideation in stroke survivors: a community-based study**

**Table S1.** Definition of predictive factors to be selected.

**Table S2.** Participant characteristics in the NHANES 2005 and 2006.

**Figure S1.** Area under curve (AUC) and Brier score results of the nomogram for predicting depression risk in stroke survivors, generated through internal validation using bootstrap resampling with 1000 iterations.

**Figure S2.** External validation results of the nomogram for predicting depression risk in stroke survivors. (A): receiver operating characteristic curve; (B): calibration plot; (C): decision curve analysis.

**Figure S3.** Area under curve (AUC) and Brier score results of the nomogram for predicting suicidal ideation risk in stroke survivors, generated through internal validation using bootstrap resampling with 1000 iterations.

**Figure S4.** External validation results of the nomogram for predicting suicidal ideation risk in stroke survivors. (A): receiver operating characteristic curve; (B): calibration plot; (C): decision curve analysis.

**Table S1.** Definition of predictive factors to be selected

| **Variables** | **Definition** |
| --- | --- |
| ***Lifestyle patterns*** | |
| Current smoking | Having smoked at least 100 cigarettes in life and now smoking cigarettes |
| Drinking | at least 12 alcohol drinks per year |
| Physical inactivity | less than 150 minutes of moderate-intensity aerobic activity per week, less than 75 minutes of vigorous-intensity aerobic activity peer week, and less than an equivalent combination of moderate- and vigorous-intensity aerobic activity |
| Sedentary time | The time usually spent sitting on a typical day not including time spent sleeping |
| Sleep duration | The time usually spent sleeping at night on weekdays or workdays |
| ***Disease and medical conditions*** | |
| Body mass index | Weight divided by the square of height |
| Waist-to-height ratio | Waist circumference divided by height |
| Hypertension | Having hypertension told by a doctor or other health professional, systolic blood pressure ≥ 140 mmHg, or diastolic blood pressure ≥ 90 mmHg |
| Diabetes mellitus | Having high blood cholesterol level told by a doctor or other health professional, fasting blood glucose ≥ 126 mg/dL, or hemoglobin A1c ≥ 6.5% |
| Hyperlipidemia | Having hypertension told by a doctor or other health professional, low-density lipoprotein cholesterol ≥ 130 mg/dL, or non-high-density lipoprotein cholesterol ≥ 160 mg/dL |
| Cardiovascular diseases | Having congestive heart failure, coronary heart disease, angina, myocardial infarction, or stroke told by a doctor or other health professional |
| Arthritis | Having arthritis told by a doctor or other health professional |
| Cancer | Having cancer or a malignancy of any kind told by a doctor or other health professional |
| Hemodialysis | Receiving dialysis (either hemodialysis or peritoneal dialysis) during the past 12 months |
| Medical insurance | Covering by health insurance or some other kind of health care plan |
| Hospitalization | Times stay in any hospital overnight or longer during the past 12 months |
| Healthcare utilization | Times seeing a doctor or other health care professional about health during the past 12 months not including times hospitalized overnight |
| Antidepressant use | Taking antidepressant medications in the past month |
| ***Functional status*** | |
| Inability to work | A physical, mental or emotional problem limiting working at a job or business |

**Table S2.** Participant characteristics in the NHANES 2005 and 2006

|  | **Data for depression** | | | **Data for suicidal ideation** | | |
| --- | --- | --- | --- | --- | --- | --- |
| **Characteristic** | **Overall (n = 118)** | **Without depression (n = 102)** | **With depression (n = 16)** | **Overall (n = 154)** | **Without suicidal ideation (n = 140)** | **With suicidal ideation (n =14)** |
| Age, years | 69.0 (61.0–80.0) | 72.5 (62.0–81.0) | 56.0 (48.5–67.0) | 68.0 (61.0–78.0) | 69.0 (61.0–79.0) | 62.0 (53.0–69.0) |
| Sex, % |  |  |  |  |  |  |
| Female | 54 (45.8) | 46 (45.1) | 8 (50.0) | 75 (48.7) | 69 (49.3) | 6 (42.9) |
| Male | 64 (54.2) | 56 (54.9) | 8 (50.0) | 79 (51.3) | 71 (50.7) | 8 (57.1) |
| Marital status, % |  |  |  |  |  |  |
| Married/living with partner | 60 (50.8) | 51 (50.0) | 9 (56.3) | 83 (53.9) | 72 (51.4) | 11 (78.6) |
| Widowed/divorced/separated | 53 (44.9) | 48 (47.1) | 5 (31.3) | 66 (42.9) | 63 (45.0) | 3 (21.4) |
| Never married | 5 (4.2) | 3 (2.9) | 2 (12.5) | 5 (3.2) | 5 (3.6) | 0 (0.0) |
| Current smoking, % | 22 (18.6) | 14 (13.7) | 8 (50.0) | 32 (20.8) | 27 (19.3) | 5 (35.7) |
| Hypertension, % | 98 (83.1) | 83 (81.4) | 15 (93.8) | 124 (80.5) | 115 (82.1) | 9 (64.3) |
| Arthritis, % | 70 (59.3) | 58 (56.9) | 12 (75.0) | 92 (59.7) | 81 (57.9) | 11 (78.6) |
| Cancer, % | 21 (17.8) | 17 (16.7) | 4 (25.0) | 29 (18.8) | 27 (19.3) | 2 (14.3) |
| Number of healthcare in the past year, % |  |  |  |  |  |  |
| 0 | 3 (2.5) | 3 (2.9) | 0 (0.0) | 3 (1.9) | 3 (2.1) | 0 (0.0) |
| 1 | 2 (1.7) | 2 (2.0) | 0 (0.0) | 4 (2.6) | 3 (2.1) | 1 (7.1) |
| ≥ 2 | 113 (95.8) | 97 (95.1) | 16 (100.0) | 147 (95.5) | 134 (95.7) | 13 (92.9) |
| Sleep duration, % |  |  |  |  |  |  |
| < 7 h | 52 (44.1) | 43 (42.2) | 9 (56.3) | 69 (44.8) | 61 (43.6) | 8 (57.1) |
| 7–9 h | 55 (46.6) | 49 (48.0) | 6 (37.5) | 71 (46.1) | 65 (46.4) | 6 (42.9) |
| > 9 h | 11 (9.3) | 10 (9.8) | 1 (6.3) | 14 (9.1) | 14 (10.0) | 0 (0.0) |
| Limited work capacity, % | 49 (41.5) | 36 (35.3) | 13 (81.3) | 71 (46.1) | 58 (41.4) | 13 (92.9) |
| Antidepressant use, % | — | — | — | 36 (23.4) | 30 (21.4) | 6 (42.9) |


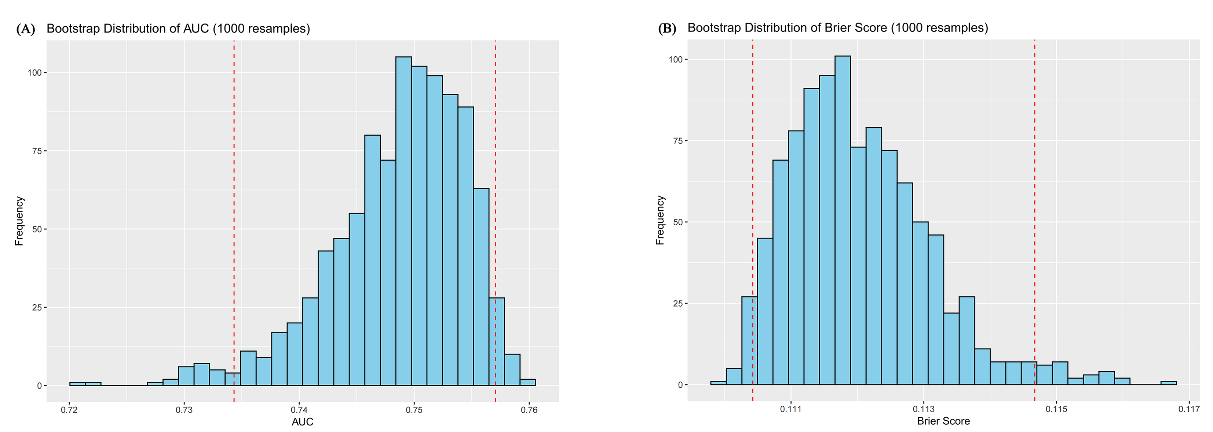


**Figure S1.** Area under curve (AUC) and Brier score results of the nomogram for predicting depression risk in stroke survivors, generated through internal validation using bootstrap resampling with 1000 iterations.


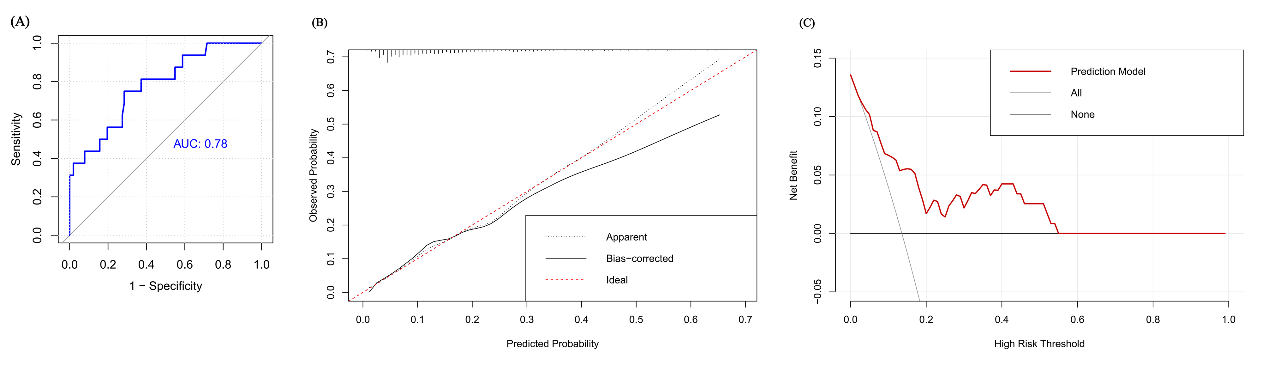


**Figure S2.** External validation results of the nomogram for predicting depression risk in stroke survivors. (A): receiver operating characteristic curve; (B): calibration plot; (C): decision curve analysis.


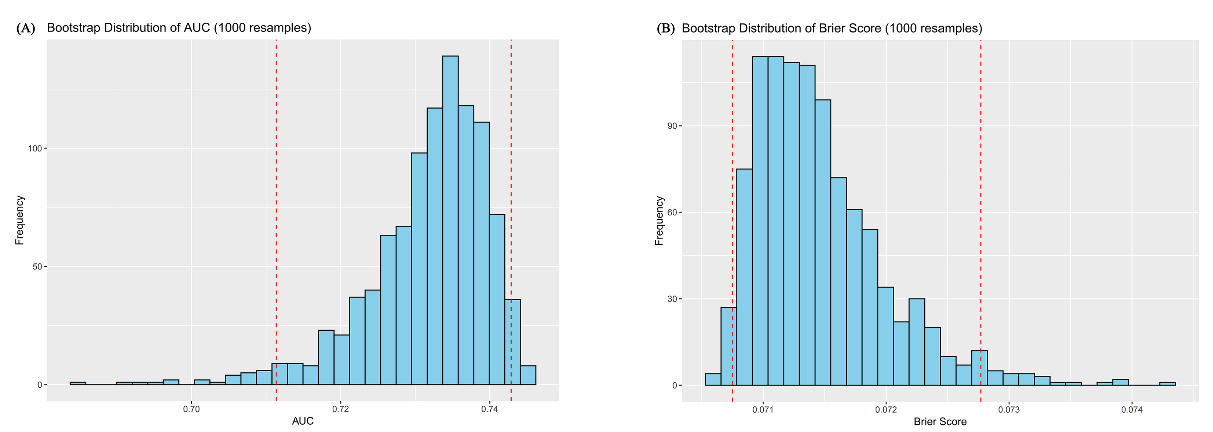


**Figure S3.** Area under curve (AUC) and Brier score results of the nomogram for predicting suicidal ideation risk in stroke survivors, generated through internal validation using bootstrap resampling with 1000 iterations.


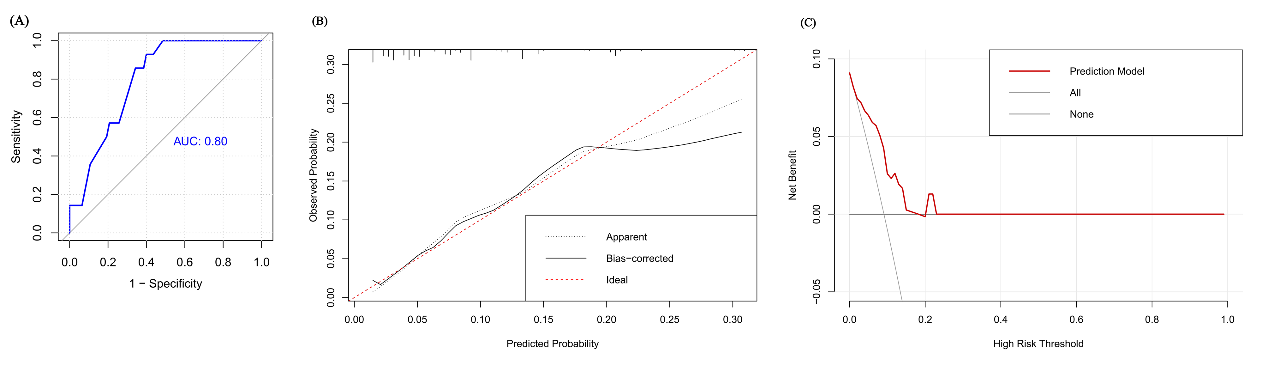


**Figure S4.** External validation results of the nomogram for predicting suicidal ideation risk in stroke survivors. (A): receiver operating characteristic curve; (B): calibration plot; (C): decision curve analysis.
